# Proteomics in Cancer Biomarkers Discovery: Challenges and Applications

**DOI:** 10.1155/2015/321370

**Published:** 2015-04-27

**Authors:** Reem M. Sallam

**Affiliations:** ^1^Clinical Chemistry Unit, Department of Pathology, College of Medicine, King Saud University, Riyadh 11461, Saudi Arabia; ^2^Obesity Research Center, College of Medicine, King Saud University, Riyadh 11461, Saudi Arabia; ^3^Department of Medical Biochemistry and Molecular Biology, Faculty of Medicine, Ain Shams University, Cairo 11566, Egypt

## Abstract

With the introduction of recent high-throughput technologies to various fields of science and medicine, it is becoming clear that obtaining large amounts of data is no longer a problem in modern research laboratories. However, coherent study designs, optimal conditions for obtaining high-quality data, and compelling interpretation, in accordance with the evidence-based systems biology, are critical factors in ensuring the emergence of good science out of these recent technologies. This review focuses on the proteomics field and its new perspectives on cancer research. Cornerstone publications that have tremendously helped scientists and clinicians to better understand cancer pathogenesis; to discover novel diagnostic and/or prognostic biomarkers; and to suggest novel therapeutic targets will be presented. The author of this review aims at presenting some of the relevant literature data that helped as a step forward in bridging the gap between bench work results and bedside potentials. Undeniably, this review cannot include all the work that is being produced by expert research groups all over the world.

## 1. Introduction

In the -omics era, the nature of high-throughput technologies, their capabilities, limitations, performance quality, and applicability are among factors determining their significance and influence not only in pure exploratory research, but also in potential clinical use.

Advances to the field of genomics and related computational tools are constantly being produced and applied in cancer-related research [1]. However, other fields are needed to complement the limitations of the genomics approach.

Proteomics-based strategy in studying diseases is considered one of the dynamic and innovative tools that could confirm, complement, or quite often provide more elaborate information beyond that obtained by other high-throughput approaches. While several genes were identified by genomics technologies to be specifically related to cancers [2], the function of such genes and the data interpretation in the context of functional networks require the power of proteomics. Moreover, although studies focusing on detecting the differential expression of mRNA have been extremely informative, they do not necessarily correlate with the functional protein concentrations. Macromolecules, in general, and proteins, in particular, are highly dynamic molecules. Mechanistically, proteins can be subjected to extensive functional regulation by various processes such as proteolytic degradation, posttranslational modification, involvement in complex structures, and compartmentalization. Proteomics is concerned with studying the whole protein repertoire of a defined entity, be it a biological fluid, an organelle, a cell, a tissue, an organ, a system, or the whole organism. Therefore, in-depth studying of proteomics profiles of various biospecimens obtained from cancer patients are expected to increase our understanding of tumor pathogenesis, monitoring, and the identification of novel targets for cancer therapy. In addition, an essential goal for applying proteomics to study cancers is to adapt its high-throughput tools for regular use in clinical laboratories for the purpose of diagnostic and prognostic categorization of cancers, as well as in assessing various cancer therapeutic regimens.

Similar to other high-throughput technologies, proteomics has been generating a vast amount of data in the form of lists of hundreds or thousands of proteins that are differentially expressed, whether increase or decrease, as a cause or consequence of ongoing physiological, developmental, or pathological events. Interpretation and analysis of such flood of information depend on building on existing data stored in constantly updated databases. Obviously, researchers have to be extra-cautious in designing their work in the first place, ensuring that good analytical tracks are being undertaken, to avoid snow ball effect and erroneous outcomes [3]. Scientifically sound analysis of the information flow as it represents complex networks and interactions of intra-, inter-, and extra-cellular environments should be the ultimate goal. Unraveling such complexity is the focus of interest for several research groups. For instance, a mass spectroscopy- (MS-) based draft of human proteome has been recently reported, which incorporated huge amount of proteomics data both from public accessed databases as well as from several research groups' work [4].

The complexity of proteomics technologies when applied to cancer research increases even more due to the current concept of cancer heterogeneity. As a matter of fact, cancer heterogeneity and biospecimen variables are considered by some researchers the most crucial and challenging point for all –omics technologies at their application in cancer studies [5].

Moreover, an integrated approach for research performed on cancers and diseases, in general, is recommended when designing studies with the intention of discovering disease biomarkers as argued by George Poste: “…The dismal patchwork of fragmented research on disease-associated biomarkers should be replaced by a coordinated ‘big science' approach” [6]. Such study designs have to comply with standardized and validated guidelines.

## 2. Mechanisms of Proteomic Changes in Cancer

Although exact causes of most cancers are not clearly defined, cancer is thought to result from a combination of genetic and environmental abnormalities. Several genomic defects have been implicated, including mutations, variation in copy number, chromosomal anomalies, and alternative splicing. One potential mechanism for the proteomic variation in cancer is the ubiquitous aneuploidy, which is defined as an imbalanced chromosomal content [7]. Aneuploid cells are thought to be under proteotoxic stress as a result of defective proteostasis; the latter is the state of dynamic equilibrium in which protein synthesis and correct folding are balanced with protein degradation. This state is a manifestation of several machineries that cooperatively ensure proper protein turnover while allowing for the conformational flexibility that is critical for proteins' biological functions. Therefore, defective proteostasis will result in not only proteotoxic stress, but also cellular dysfunction and subsequent pathologies [8]. Recent findings have shed some light into the yet-not-fully-understood mechanisms underlying the association between aneuploidy, proteotoxic stress, and abnormal cellular proliferation and tumorigenesis [7]. However, this association is still a matter of controversy and is lacking straightforward relationship pattern; for instance, an extra chromosome that results in increased gene expression and a theoretical increased protein production is not necessarily translated into an actual elevation of circulating protein levels, since there is high possibility of overwhelming the cellular protein folding apparatus, leading to chronic protein misfolding and subsequent protein degradation. It is proposed that certain proteins, such as various kinases and multimeric protein complexes, have increased requirements for the cellular protein folding apparatus, and hence they are more susceptible to misfolding than others. This and other relevant examples are comprehensively reviewed by Donnelly and Storchová [7]. Emerging evidence linking aneuploidy, defective proteome, and cancer development is of obvious significance as it provides potential for treating aneuploid cancer cells using suitable antineoplastic agents targeting the proteostatic machinery [9]. This will be discussed in more details later.

Another potential mechanism for proteomics changes in cancers is the consequence of defective protein structure and hence function. Mutations in cancer-associated genes can be manifested in defective protein structure. These defects can exert their deleterious impact through changing protein stability and causing the protein to be more susceptible for degradation; changing the protein's functional site residues; or changing the affinity controlling protein-protein interactions [10].

Genomic and proteomic changes in cancer could be further followed up by the recently emerged field of “interactome profiling” focusing on network-centered approach, that is providing an enormous amount of data representing protein interactions and the influence of protein structures. This is reviewed recently by Gulati and coworkers and is beyond the scope of the current review [11].

## 3. Cancer Biomarkers' Applications: Challenges and Recommended Solutions

### 3.1. Cancer Heterogeneity

The current concept of cancer heterogeneity and biospecimen variables is considered by some researchers as one of the most crucial and challenging points for proteomics as well as for other -omics technologies, at their application in cancer studies. Recently, intratumoral heterogeneity was examined in invasive breast cancer, comparing biospecimens obtained by intraoperative image-guided, core-needle biopsies to surgical biopsies taken from the center and the periphery of cancer breast. Proteomics techniques undertaken in that study have demonstrated that even though most biomarkers studied did not manifest significant intratumoral heterogeneity, protein and phosphoprotein levels were affected by biospecimen type, as well as by other preanalytic variables, including surgical manipulation and the duration of cold ischemia [5]. A recent approach to circumvent the challenge of tumor heterogeneity and to extract meaningful data from formalin-fixed tissue blocks has been suggested, combining matrix-assisted laser desorption ionization (MALDI) with imaging (MALDI imaging mass spectroscopy; MALDI-IMS). This approach is unique as it allows proteomics-based studies to provide both patient-specific and cancer-specific information as a means for biomarker discovery and cancer tissue classification. It also provides morphology-based proteomics analysis for cancer tissue [12]. In addition, studies using MALDI-IMS analysis of specific cancer tissues generate peptide reference datasets to facilitate peptide identification in future studies on the same cancer type. However, several technical challenges still exist including low signal to noise ratio and low mass accuracy [13]. In a recent work studying prostate cancer, Shipitsin and coworkers have developed a biopsy simulation procedure by tissue microarrays aiming at exaggerating prostate cancer tissues' variation that is expected in clinical practice. Their approach has provided a useful model for predicting cancer aggressiveness through reliable biomarkers, regardless of sample variation [14].

### 3.2. Cancer Early Detection

Detecting cancer at an early stage, when there is a better chance for its treatment, is a real challenge to the scientific and medical communities as most clinical blood biomarkers assays do not have the required sensitivity and specificity necessary for that purpose. In an interesting approach focusing on ovarian cancer, Hori and Gambhir have recently developed a mathematical model looking at the estimated time at which ovarian cancer can be detected by measuring the amount of the cancer antigen 125 (CA 125) shed from the tumor during its growth. Surprisingly and despite the reported sensitivity of the CA 125 measuring assays, the authors reported that a tumor could grow unnoticed for more than 10 years and reach a size of more than 2.5 cm before becoming detectable. This mathematical approach might yield similar finding in other tumor types, and the model can be extended to virtually any solid cancer and associated biomarkers, according to the authors' suggestions [15]. Nevertheless, a lot of debate has emerged regarding the applicability of this approach in other types of tumor and the sort of assumptions used in its calculation [16]. This example illustrates a unique approach to test the applicability of circulating biomarkers' assays in early cancer detection, cancer prognosis, and therapeutic response and monitoring. Combining panels of circulating biomarkers, rather than a single molecule, with newly developed or newly updated technologies such as imaging procedures might be more informative in terms of early diagnosis, accurate assessment of the prognosis, and response to therapy in cancer patients [16, 17].

### 3.3. Protocols for Developing Tumor Biomarkers

More than a decade ago, several research groups have formulated multisteps strategies for developing tumor biomarkers. Hammond and Taube's phased approach involved the following steps/phases: the biomarker discovery, the development of an assay system, the performance of preliminary analysis for the biomarker's clinical potential, the standardization and assessment of the biomarker's measuring assay, and finally the validation of that assay for clinical use [18]. Despite the strict step-wise analytical criteria of this strategy, preanalytical issues were not sufficiently addressed. About the same period of time, Pepe and colleagues suggested another strategy that focused on the need for accurate definition of the study's aims and its outcomes together with strict criteria for specimen selection, sample size calculation, and experimental methods [19]. Several years later, the same group has suggested a more rigorous study design for the development of tumor biomarkers, emphasizing that the described design would maintain a high research quality and improve the possibility of obtaining a clinically promising biomarker ready for subsequent rigorous scrutiny [20]. Common biases that plagued the process of biomarker discovery research were claimed to be avoided if this design, which was called “nested case-control study design” was strictly followed. The design included prospective collection of specimens before outcome ascertainment from a case-control study cohort that is relevant to the clinical application under study, and blind assessment of the biomarker in specimens obtained from randomly selected case and control subjects. The authors described various aspects of their design in relation to the clinical context, biomarker performance criteria, biomarker test, and study size [20].

### 3.4. General Guidelines for a Good Study Design for Biomarkers' Discovery

In order to plan a good study design for cancer biomarkers discovery, several aspects have to be meticulously tackled. This was reviewed in more details elsewhere [21, 22] and is summarized in the following section. Firstly, careful planning starting with the formulation of a research question supported by convincing evidence for its importance and relevance to a clinically pressing problem. A rational choice of the most suitable analytical tests to approach this research question is of equal significance. The performance characteristics for such test(s), in terms of specificity, sensitivity, and positive and negative predictive power, should be appropriate for the experimental design and clearly described. In addition, the verification and validation strategies of the method(s) performed and the clear and detailed description of the samples' nature, collection, and storage protocols have to be openly defined. Details of the samples' source, as the subjects' age, gender, disease stage, medications taken, and lifestyle are necessary to be highlighted as well. Furthermore, in cancer tissues' biomarkers-related research, the sampling procedures are of critical importance due to their heterogeneity. Therefore, collecting a representative sample is important in order to obtain reliable data. Likewise, sample size calculation is a crucial component of the study coherence and if carefully conducted will average out sample heterogeneity. Moreover, protocols of executing the experiments should maintain basic and critical points, such as incorporating proper blank(s), positive and negative control samples, and reference compound(s) within* each* run for the analytical procedure. Details of the quality performance of instrumentation and their calibration are equally important for the procedure's validation. Collectively, every step in the study design and execution has to be clearly described in sufficient details to allow for reproducing the work and/or comparing the data.

The scientific communities have been working diligently to standardize the procedures of proteomics-generated data optimum utilization. Useful data repositories have been constructed such as Panorama (https://panoramaweb.org/) that, together with portals for proteomics assays involved in targeting cancer-related proteins and peptides, will enable researchers interested in specific protein or peptide to obtain the standard operating procedures (SOPs) for those assays, their quality assessment, and validation proofs [22].

## 4. Proteomics Techniques Used in Cancer Research

Research studying protein alterations in cancer existed for more than 70 years [23]; however it was only in the last 3 decades or so that recent proteomics technologies have been extensively utilized in deciphering protein differential expression in human cancers [24]. Various approaches have been carried out, taking advantage of the recent analytical techniques and advanced bioinformatics. In general, two main proteomics tracks can be undertaken, the “shotgun” or “bottom-up” methods and the targeted proteomics methods. A recent set of “best practices” for MS-based assay development using the concept of “fit-for-purpose” was recently published following a workshop that was held in mid-2013 in the United States of America's National Institutes of Health (NIH) with representatives from different institutes involved in the development and/or utilization of targeted proteomics assays [22]. The following section starts by briefly describing basic techniques such as 2D gel electrophoresis, difference in gel electrophoresis (DIGE), and MS, followed by introducing more recent technologies and combined applications such as protein microarray and combined proteomics and imaging methods.

Polyacrylamide gel electrophoresis (PAGE) and isoelectric focusing (IEF) have been the basis for the 2D PAGE techniques resolving proteins based on their molecular mass and isoelectric points, respectively. This approach has been frequently applied to analyze cancer cells proteins for more than 2 decades [25] and is still in use [26]. Further advancement in this approach has been the result of introducing fluorescent dyes and in-gel comparative proteomic analysis in the technique of 2D-DIGE. This is usually coupled to protein spot analysis by fluorescence gel scanners, spots' picking, and enzyme digestion, followed by identification by one of the MS-based available techniques.

Advancements in MS resulted in optimal performance in the low mass range of proteins. In-depth profiling of plasma and other biofluids proteomes results in identification of proteins that span more than six logs of protein abundance. As such, it has been the method of choice in many cancer applications [24]. To detect low-abundance proteins, an initial samples' preclearing step might be performed to remove the high-abundance proteins, such as albumin and immunoglobulins. However, this carries the risk of depleting the samples from the low-abundance and low-molecular weight proteins that are bound to the circulating carrier proteins. The latter have been demonstrated to act as a reservoir storing diagnostic information within the accumulated bound low-molecular weight potential biomarkers [27]. Incorporation of bead-based immunoassays may also be used to better identify low abundance proteins [17].

MS use in protein analysis has undergone several stages of technical advancement and improved instrumentation efficiency. MALDI-MS [28] and electrospray ionization (ESI) MS, combined with advancements in protein fractionation and separation, as liquid chromatography (LC) and gas chromatography (GC) and labeling techniques, are examples of such technological developments. This has been thoroughly reviewed in several articles [24, 29–31]. More recently, proteomics approach has been extended to involve studying of epigenetic processes in cancer research. The use of MS-based proteomics in studying various aspects of chromatin biology and in evaluating specific histone posttranslational modifications resulted in the discovery of chromatin-associated proteins and multisubunit complexes that can be considered epigenetic biomarkers with future potential in cancer diagnosis and therapy. This has gained a wide attention and was recently reviewed by Bartke et al. [32].

Microarray is considered one of the most exciting developments in high-throughput technologies. Simultaneous measuring of the expression of thousands of genes (gene microarray) or proteins (protein microarray) and detection of genomic or proteomic biomarkers, respectively, that are tightly linked to cancer development and/or progression have revolutionized the cancer research studies. Recently, such technologies have been applied to study a relatively uncommon category of cancer patients who are presenting with metastatic cancer without any obvious anatomically detectable primary tumor, the so called cancer of unknown primary or CUP [33].

In addition, the targeted proteomic approach of selected reaction monitoring (SRM) has been developed and widely applied, for instance, to detect mutant proteins in the colorectal cancer tissue and in the fluid obtained from potential precancerous pancreatic cysts [34]. Other recent approaches have been described in the literature, including a multiplexed microfluidic immunohistochemistry-/immunocytochemistry-based quantitative proteomics profiling of cancer samples [35].

Combining proteomics and imaging-based methods has been recently described. Shipitsin and coworkers were able to identify a panel of 5 protein biomarkers for prostate cancer lethality using an automated, integrated quantitative multiplex immunofluorescence in situ imaging approach [36]. Such combination is thought to produce more clinically representing data in terms of the actual* in vivo* environment where the active proteins exert their functions. This is because such approach was designed to measure the levels and activity status of protein biomarkers in defined intact tissue regions, avoiding the need to lyse the tissues of interest that is commonly performed in the traditional proteomics approaches. Wider range of applications and comparative studies to more established proteomics approaches is still in progress. In a different context, integrating proteomics and imaging tools to gain more insight into the pathogenesis of cancer progression and penetrability at the molecular level is recently experimented. An article describing such mechanistic-oriented approach was recently published by Oh and colleagues [37]. This group used their advanced integrative tools to study caveolae at the blood-solid tumor interface* in vivo* aiming to reveal molecular portals to infiltrate solid tumors of mammary, prostate, and lung origins. They were able to reveal a transvascular pumping system and define some of its component proteins, as caveolin 1 and annexin A1, that are affecting tumor uptake of various agents. Such approach will probably get a large scale attention as it can be applied to assess the effectiveness of therapeutic agents based on their ability to cross the biological barriers* in vivo* and find their way into the solid tumors.

## 5. Examples of Proteomics Research Applications in Various Cancer Types

In various types of cancer, the biomarkers discovery is expected to improve one or more of the following critical applications: early diagnosis and prognosis and monitoring of disease progression, its response to therapy, and its recurrence. High-throughput hypothesis-generating methods have revealed hundreds to thousands of cancer associated proteins (CAPs). This implies that hundreds to thousands of potential protein biomarkers have been suggested in the literature and are awaiting proper validation. It is only after validating these molecules that they can be considered for application in diverse clinical setting such as diagnosis, prognosis, disease staging, and patients' categorization. This is a critical aspect in translational cancer research [38, 39]. Classically, hypothesis-testing has been performed using antibody-based methods, such as enzyme-linked immunosorbent assays (ELISA). However the limited availability of validated ELISAs and their high cost and time-consuming nature, together with the technical challenges of assay multiplexing, all have been obstacles hindering the use of these assays in validating the rapidly evolved lists of potential cancer protein biomarkers [40].

Due to the lagging in high-throughput hypothesis-testing methods, these CAPs cannot yet be applied in clinical setting. Therefore, a pressing need for accurate, precise, and sensitive validation assays has been the driving force for an ongoing extensive recent research. One promising track has generated selected reaction monitoring (SRM) assays of targeted proteomics. SRM assays have been recently developed and refined for many human CAPs that are functionally related to cancer driver mutations. They have been used to measure the detectability of target proteins in the circulation or urine and have resulted in reproducible quantification across cohort of cancer patients' samples. Therefore, these assays are thought to represent a valuable resource for accelerating and planning biomarker verification studies [41].

The following section describes some of the proteomics research outcomes in three of the most-studied cancers: lung, breast, and ovarian cancers. A more detailed discussion will be presented for the ovarian cancer aiming at emphasizing few points of critical significance. For instance, various perspectives in approaching the subject of cancer biomarkers, the need to standardize and optimize study design, preanalytical and analytical assays components, and strict validation strategies are among the points to be discussed in more detail for ovarian cancer. A description of how proteomics has helped in clarifying the ovarian cancer markers for carcinogenesis, cancer progression, diagnosis, prognosis, and targets for therapeutic treatment will be presented as well.

### 5.1. Lung Cancer Biomarkers: Implications from Proteomics

Lung cancer signatures in plasma have been studied both in mouse models and in human with data implying concordance between findings in both species [42]. Circulating levels of EGFR as well as other biomarkers as SFTPB and WFDC2 were significantly different in lung cancer cases relative to control. As with the case of other types of cancer, finding a marker or a panel of markers, which has a screening power, if measured in prediagnostic biological sample is an important goal, as it carries the potential for application in early detection or even screening strategies of lung cancer in addition to the potential use for monitoring subjects diagnosed with the disease [42]. Unfortunately, this goal is not yet achieved. As mentioned in previous sections, recent technologies integrating proteomics and imaging tools are being used, with promising results, to gain better insight for the pathogenesis of lung cancer at the molecular levels. This is expected to improve our understanding of the effectiveness of anticancer therapies in terms of their ability to be successfully delivered in the required dosage to solid tumors [37].

### 5.2. Breast Cancer Biomarkers: Implications from Proteomics

Proteomics approach in studying breast cancer has also been progressing and yielding promising findings with both diagnostic and therapeutic applications. An example of combined* in vitro* and* in vivo* approaches involving deep analysis of cultured breast cancer cell lines was obtained from tumors of defined breast cancer stages and validated using human breast cancer tissue. This approach has demonstrated that the tumor stage-specific proteomic signatures extracted from the* in vitro* study were validated on tissue microarrays. Transformed cells showed proteomic signatures characterizing the loss of tissue architecture and the cellular metabolic changes [43]. Remarkably, recent work has shown that the plasma proteome in breast cancer also indicates the tumor microenvironment-derived proteins involved in a number of innate physiologic processes such as wound repair, immune response, and tissue remodeling [44].

### 5.3. Ovarian Cancer Biomarkers: Implications from Proteomics

Recent epidemiological studies have demonstrated that ovarian cancer remains a serious condition that is considered the most lethal gynecological malignancy [45]. Unfortunately, very few cases are diagnosed at clinically early stages, and the vast majority are diagnosed at late stages with the tumor already spread distantly [45]. Moreover, due to the low prevalence of ovarian cancer, no screening test is available for population screening. In fact, with the condition's low prevalence, a screening test has to be of an extremely high specificity to possess an acceptable positive predictive value [46]. The condition is pathologically not-very-well understood, and, few years ago, the use of molecular profiling has confirmed that ovarian cancer represents a heterogeneous class of diseases that are sharing a common organ [47]. Therefore, there is a pressing need for discovering novel biomarkers to improve the outcomes of such a serious disease.

Ovarian cancer-induced altered biologic processes are expressed as aberrant molecules that belong to various biochemical families, such as DNA, mRNA, proteins (and related subfamilies as glycosylated proteins, peptides, and autoantibodies), and metabolites. The recent technology breakthroughs in genomics and proteomics fields have positively influenced our understanding of the pathophysiology of the disease.

To date, only 2 individual circulating biomarkers, CA 125 and human epididymis protein 4 (HE4), are approved by the American Food and Drug Administration (FDA) for monitoring treatment and detecting recurrence in ovarian cancer patients. In addition, the FDA has recently approved two algorithms to be used clinically as a supplement for decision making for preoperative adnexal mass patients [46].

Several research groups worldwide focus on studying the altered biology in ovarian cancer and to discover promising molecular mediator(s) as biomarkers or as therapeutic targets, using proteomics tools. Such tools target, beside the proteins repertoire, other related biochemical entities, for example, the glycosylated proteins (glycomics), the low molecular weight peptides (peptidomics), the metabolites (metabolomics), and the antitumor antibodies (immunoproteomics). These entities have been reviewed recently by Leung et al. [46]. Mechref and coworkers have described major advances in both preanalytical separation methods and MS that allowed for increasingly comprehensive characterization of glycosylated proteins repertoire (the glyome) and cancer-specific glycoproteins in various types of cancers including ovarian cancer [48]. Although the detection and characterization of aberrantly glycosylated proteins in biospecimens still face technical challenges, recent advances in MALDI-MS and in the preanalytical enrichment methods such as peptide-N-glycosidase digestion and chromatographic separation have enabled glycoproteomics techniques to positively add to the list of cancer-specific glycoproteins [49–51]. Glycosylation as a posttranslational modification is described as heterogeneous, structurally complex, widespread, and cell- and protein-specific process. Therefore, in studying the cancer-specific glycans, researchers are faced with both technical limitations and uncertainty in the biological interpretation. Examples of the technical challenges are the heterogeneity of the glycans resulting in a collection of glycoforms and isomers for each glycoprotein and the limited ability of most current proteomics technologies to precisely differentiate these forms and isomers. Moreover, following the discovery of candidate glycan biomarker(s), there should be reliable quantitative validation assays, with good specificity for the glycan epitope, as well as good sensitivity. Currently, there have been trials to develop such assays, using lectin or antibody capturing technology; however these are not yet sufficiently available for strict validation. To complicate the matters even more, researchers struggle to determine the biological implications for the aberrant glycoproteins' profiles in cancer states. In the context of ovarian cancer, it is not clear whether the components of glycomic profiles published in the literature are unique to this cancer or, alternatively, are a consequence of cancer-related metabolic defect(s). Therefore, more rigorous investigations are clearly needed in this field [52].

The study of the global metabolites population in biospecimens, the metabolome, by MS-based assays has been increasingly utilized in the field of cancer biomarkers discovery. Biological fluids as urine and serum or plasma are the usual specimens used. Urine specimens are sometimes preferred in proteomics and related technologies for the biomarkers discovery over serum or plasma. The reasons for this preference include the relatively low total protein concentration in normal urine and the noninvasive nature for urine sample collection. Urine sample is relatively free from high molecular mass proteins making it less complicated than the serum/plasma samples [53]. Proteomics technologies as ultraperformance LC quadrupole time-of-flight MS (UPLC-Q-TOF MS), hydrophilic interaction chromatography, and reversed-phase LC MS were able to identify several metabolites in the urine of ovarian cancer patients as compared to healthy control subjects. Interestingly, some of these metabolites were discriminatory between early and late clinical stages of those patients [54, 55]. Recently the metabolomics profiles of plasma samples obtained from epithelial ovarian cancer (EOC), benign ovarian tumor (BOT), uterine fibroid, and healthy controls using UPLC were published. Fifty-three metabolites were identified in this work as specific biomarkers for EOC. Again, these metabolites were able to discriminate EOC from BOT and uterine fibroids, as well as early-stage from late-stage EOC. The critical analysis of the aberrant metabolites has identified unique metabolic pathways that were disturbed in cancer cases, namely, those of phospholipids metabolism, tryptophan catabolism, and fatty acid *β*-oxidation. These findings are expected to increase our understanding of ovarian cancer pathophysiology [56]. Despite the noticeable advances in this approach, a number of confounding variables are still hindering the introduction of metabolomics for full clinical application. Technical limitations include the biases related to preanalytical factors as sample collection and storage conditions. Biological limitations involve the unstable nature of metabolites that may be extensively transformed during transition from the cancer site to the biospecimen collected or even after collection. Moreover, other confounding factors include the subjects' age, smoking habits, sleep patterns, and lifestyle. Hence, standardized and robust protocols are needed to eliminate such biases and to allow for assay's precision [46].

Ascites fluid has been studied as a source for proteomics and metabolomics potential biomarkers in ovarian cancer, with an advantage over plasma or serum due to its close proximity to the site of the tumor. Comparing malignant ascites with cirrhosis ascites' metabolomes has identified 41 metabolites that differed significantly between both pathologies. Detailed analysis of these metabolites has revealed that most of the cancer-specific metabolites belong to signaling pathways. Similarly, proteomic analysis has identified even more molecules discriminating the ovarian cancer from cirrhosis ascites. Interestingly, spliceosomal proteins and RNAs were found in the ovarian cancer ascites, a finding suggesting that these molecules might play an essential role in intercellular communications between cancer cells [57].

More recently, low-molecular weight proteomics or peptidomics has been used in studying biospecimens as blood, urine, ascites, or even tumor tissue, seeking to identify unique biomarkers for the ovarian cancer [58–60]. This approach, although still in the beginning, is promising, and, if standardized, is suggested to complement the conventional proteomics approach as it reflects the cancer-related protease activity. However, the lack of standardized protocols and of robust quantifying validation assays is hindering its widespread use.

The last decade has also witnessed a novel approach in cancer biomarkers discovery that targets identifying cancer related populations of antitumor antibodies, the so called immunoproteome [61, 62]. However, similar to peptidomics, this approach is still lacking appropriate validation assays before any applications for the identified potential biomarkers can be suggested.

As mentioned above, the proteomic profiling of plasma is quite challenging due to the high dynamic range of protein concentration which makes it hard to identify low-abundance proteins. Some researchers have turned their attention into more proximal biofluids such as the ovarian-tumor-tissue-interstitial fluid as more promising sample sources [63]. However, because a good sample for clinical use should be easily accessible, results of the biomarkers candidates produced by this approach must be assessed and rigorously tested in more clinically relevant body fluids, such as serum, urine, or saliva, before being considered as a tumor specific biomarker [64]. As previously mentioned, screening assays for ovarian cancer among healthy individuals are lacking and are indeed seriously needed due to the aggressive course of the late-diagnosed disease. This is not-yet-feasible despite the ongoing active research. For instance, Moore and coworkers have combined an immunoassay for CA 125 with a proteomic approach: surface enhanced laser desorption ionization time of flight MS (SELDI TOF MS) to assess and quantify a panel of 7 biomarkers (apolipoprotein A1, truncated transthyretin, transferrin, hepcidin, *β*-2-microglobulin, connective tissue activating protein III, and inter-alpha trypsin inhibitor heavy chain 4), aiming at improving the specificity and sensitivity of detecting EOC in preclinical cases using prediagnostic serum samples [65]. The experimental design was based on previous published work by the same research group demonstrating that using this combination in postdiagnostically collected sera has increased the sensitivity for detecting ovarian cancer beyond CA 125 alone [66]. However, addition of these biomarkers to CA 125 failed to enhance the sensitivity for preclinical diagnosis [65]. The need is still pressing for a biomarker or a panel of biomarkers that could be relied on in screening for ovarian cancer. Strong and evidence-based data can be a real challenge but should not be rushed to produce. More than a decade ago, serum proteomic patterns capable of discriminating normal subjects from ovarian cancer patients were published and got a lot of attention, both in the scientific community and among the decision makers and sponsoring agents [67]. However, more scrutinizing analysis for those findings has demonstrated a defect in the experimental design that prevented their reproducibility and that emphasized on the critical importance of a good design to obtain reproducible data [3].

Essentially, extensive advances in the traditional proteomics and its more recent related technologies are producing vast amounts of data for ovarian cancer. Appropriate standardization and validation of assaying these potential biomarkers, whether individually or in combinations, are critical, prior to introducing them as clinical determinants, for screening, diagnosis, prognosis, and monitoring for the response of treatment or for recurrence.

From the previous section, it is evident that results obtained from proteomics and related technologies contributed positively in the past and are expected to remain capable of doing so in the future, to obtain better understanding for ovarian cancer. The following discussion highlights few examples of various aspects of this contribution.

1. Ovarian cancer pathogenesis: Proteomics has resulted in better insight into the molecular bases of ovarian cancer pathogenesis. For instance, overexpression of particular signaling pathways' molecules within ovarian cancer cells have been described in the literature as a possible mechanism underlying or associated with the condition. Signaling pathways involved in cancer cell differentiation, survival (proliferation or apoptosis), migration, and metabolism are most commonly affected during the pathogenesis of cancer ovary. Examples of these pathways include the lysophosphatidic acid, the phosphatidylinositol 3-kinase, NF *κ*B, the MAPK, and the vascular endothelial growth factor signaling pathways [68, 69]. These findings provide essential information about potential diagnostic and prognostic markers, as well as therapeutic targets for future pharmacotherapeutic-oriented ovarian cancer research. Furthermore, recent publications demonstrating the results obtained from a large Gynecologic Oncology Group trial are producing promising data. For instance, specific patterns of glycans were found to be discriminatory in distinguishing epithelial ovarian cancer and low malignant potential ovarian tumor cases from normal individuals. The candidate glycan biomarkers demonstrated sensitivity and specificity high enough to suggest further in-depth validation prior to using them as diagnostic markers for early detection of ovarian cancer [51].
2. Etiologically, ovarian cancers can be sporadic or hereditary. Risk factors that increase women's susceptibility to ovarian cancers include genetic mutations as those reported in BRCA1 and BRCA2 genes and the mutations in the DNA mismatch repair genes characterizing Lynch syndrome [70]. Proteomics techniques can be performed to detect the profile(s) characterizing these mutations. For instance, a proteomic signature predicting the malignant transformation of conditions with high risk of developing ovarian cancers, such as ovarian endometriosis and pelvic inflammation during ovarian carcinogenesis, is of great significance [71]. Increasing awareness of the hereditary aspect of gynecological tumors such as breast and ovarian cancer has resulted in a remarkable interest in screening populations at high risk for these malignancies. Specialized cancer centers and institutes have been formulating programs aiming at multidisciplinary coordinated approach for evaluating women with high risk of breast and ovarian cancers, organizing appropriate clinical care, updating relevant recommendations and guidelines, providing support to patients, and facilitating enrollment in appropriate research studies and registries [72]. Proteomics analysis has been performed for samples obtained during the surgical procedure of risk-reducing bilateral salpingooophorectomy (RRBSO) that is undertaken for women of high risk category. LC/MS MS and protein network database algorithms were used to evaluate the proteomic profiles characterizing the pathological changes in this group of high risk women. Few years ago, a high-throughput workflow for analyzing the proteomes of pelvic tissues (peritoneal, fallopian tube, and ovarian surface epithelial samples collected at the time of this surgery) has been described. The aim for this approach was to discover novel biomarkers that could have predictive or diagnostic value in the pelvic tissues to identify precancerous and cancerous proteomic changes of high risk deleterious mutations carriers [73].
3. Ovarian cancer progression: The transition of benign ovarian tissue into its early malignant transformed state is such a critical step that should be extensively studied aiming to obtain a descriptive profile for it, since, as already mentioned, the ovarian cancers have notoriously poor prognosis and a highly aggressive clinical course. Proteomics technologies have been involved in following up ovarian cancer progression by evaluating the protein expression profiles in cancers of different clinical and pathological stages and in normal ovarian epithelium tissues. By performing 2D electrophoresis combined with MALDI-TOF/TOF techniques, Li and coworkers have identified 54 aberrantly expressed proteins in serous ovarian cancers. The expression of one of those proteins, the glia maturation factor beta (GMFB), was further analyzed in large cohort of patients with various stages of ovarian cancers and was found to be significantly increased as compared to normal, benign, or borderline ovarian tissues. The statistically significant positive correlation between the expression of GMFB and the FIGO staging of the tumor, and the association between this protein's expression and a poor disease-free survival and overall survival, together with the multivariate analysis results, all have suggested that this protein is an independent prognostic factor for disease-free survival and overall survival in the studied serous ovarian cancer patients [74]. Other research groups have performed slightly different approaches on various biospecimens. For instance, combining shotgun proteomics and SRM MS, Elschenbroich and colleagues have published the results of in-depth proteomics analysis of ovarian cancer ascites as compared to ascites from benign ovarian tumors. They have designed an analysis pipeline that included discovery-based proteomics, bioinformatics, and targeted proteomics quantification of the detected cancer biomarkers candidates [75]. Combined 2-DE and MS/MS analysis has been used to study the ovarian cancer tissue, interstitial fluid, and peritoneal effusion, as compared to normal tissue and fluid, in specimens obtained surgically [76]. This comparative analysis has revealed differential expression of six proteins that are involved in cell cycle progression and apoptosis, as well as in signal transduction pathways. One of those proteins, the calgranulin, was reported to be significantly overexpressed in all pathological samples and to represent a potential diagnostic and/or prognostic biomarker. Other studies have reported changes in N-linked glycan structures and their expression as diagnostic signature in ovarian cancer patients [50]. A shotgun quantitative proteomic evaluation of benign and malignant epithelial ovarian tumors as compared to normal tissue, using iTRAQ technology with LC-MALDI-TOF/TOF and LC-ESI-QTOF MS/MS was published two years ago. The PI3K/Akt signaling pathway was reported as a significant pathway capable of discriminating the clinicopathologically different tissues studied [77]. More recently, MS analysis of the secretome from* ex vivo* coculturing of ovarian cancer cells and peritoneal cells to detect proteomic markers of their interactions was suggested to reflect the metastasizing nature of ovarian cancers. A protein, Mucin 5AC was suggested as a potential biomarker for the invasiveness of ovarian cancers since its expression was significantly elevated in the ovarian-peritoneal cells coculture as compared to monoculture of each type of cells [78]. Furthermore, overexpression of class III *β*-tubulin within the ovarian tumor microenvironment was recently demonstrated to have prognostic power predicting poor overall survival in patients treated with neoadjuvant chemotherapy [79].
4. Targets for therapeutic means: A rare histologic subset of ovarian cancer, clear cell ovarian cancer, is known to have low survival relative to other types of ovarian cancers. Genomics and immunohistochemical studies have demonstrated similar gene and protein expression profiles to clear cell cancers in other organs, specifically the kidney and uterus. Therefore, it might be recommended to consider therapeutic approach of this serious cancer histotype based on the protein expression profile, rather than on the organ affected [80]. Few years ago, Anglesio and coworkers have demonstrated that women with clear cell ovarian cancer had shown a positive response to Sunitinib, a drug used with relatively successful outcome in patients with renal cancers [81]. Additional new perspectives for novel targets in ovarian cancer therapy are being examined utilizing data obtained from various high-throughput technologies [82].

## 6. Can Proteomics Research Findings in Cancer Be Translated into Clinically Oriented Research?

As already mentioned, massive applications of recent -omics technologies in cancer research have started since the last century and have been constantly evolving so far. These have been translated into genomics and proteomics cancer signatures. The translation of biomarker discoveries into potential anticancer agents is highly dependent on the quality of data generated, which is influenced by several factors as mentioned above [83]. Wilhelm and colleagues have recently reported an MS-based draft of human proteome. Among their findings of human proteome expression, they confirmed high levels of expression of functional proteins in relation to specific cancer. For instance, the protooncogene EGFR, which was discovered in the eighties of the last century [84], was recently found to be highly expressed in a confined manner to certain cancerous tissue as in breast cancer. Beta-catenin, a member of the Wnt signaling pathway, was also highly expressed in colon cancer cells, where it participated in the development of the malignancy [4]. These findings and others represent a rich source of information and a platform, based on which researchers can design projects aiming at discovering novel anticancer agents. The following section summarizes information from 2 research groups working on example of such agents, the EGFR kinase inhibitors and the heat shock protein 90 (Hsp90) inhibitors.

### 6.1. EGFR Kinase Inhibitors

Studying the cellular mechanisms of cancer in general and of drug action in particular has been a hot area in proteomic cancer research. This area holds a promising outcome of clinical significance and hence the hope of moving cancer proteomics from bench to bed side [24]. Using cancer cell line panel developed by the National Cancer Institute (NCI) as a model system for different tissue types and genetic diversity of human cancers, and analyzing the massive amount of information obtained by bioinformatics, Moghaddas and coworkers have shown a strong cell line clusters based on tissue type. Hundreds of differentially expressed proteins were demonstrated in this model system, which are potential biomarkers for different tumor properties. Moreover, by integrating their proteomic data to the publicly accessed transcriptomic data for this model system, the authors have shown consistency between mRNA and protein expression. They were also capable of demonstrating that protein expression can be correlated to many FDA-approved anticancer drug response, both drug sensitivity and resistance [85]. Of special importance as anticancer drug targets are various families of cellular protein kinases. Kinases represent important oncogene classes and are key players in intracellular signaling; subsequently their differential expression and/or functional dysregulation can be a cause or consequence of tumorigenesis. Therefore, not surprisingly, kinases are important anticancer therapeutic targets [86, 87]. The EGFR kinase inhibitors erlotinib and lapatinib have been used in cancer therapy. Recently, proteomics approach in cancer cell lines using elastic net analysis has been utilized for the identification of markers for drug sensitivity (positive-effect-size) or resistance (negative-effect-size) [4].

### 6.2. HSP90 Inhibitors

Hsp90 is a molecular chaperone that is essential for the correct folding, stability, and hence functions of many proteins. As such, it is part of a system that functions in both physiological and pathological states [88]. Cancer cells are considered chaperone addict, since they have special requirement for the protein folding machinery components to deal with the surplus of proteins being synthesized. The significance of targeting Hsp90 in cancer therapy lies in the nature of its clients, since many of them belong to the family of oncogenes, including tyrosine kinases, transcription factors, and cell cycle regulatory proteins. Therefore, inhibiting Hsp90 leads to degradation of such proteins through the proteasome machinery. The use of Hsp90 inhibitors in treating cancer has been promising in certain solid tumors as well as in hematological malignancies. This has been recently reviewed by Garcia-Carbonero and coworkers [89].

## 7. Conclusion and Perspectives

Proteomics approach in studying many diseases including cancer is producing data that complements those produced by other high-throughput technologies. Such technologies should aim beyond the mere generation of lists of differentially expressed macromolecules and their derivatives, as a cause or consequence of the studied pathology. For instance, careful interpretation of proteomics data has shed some light on the underlying mechanisms leading to cancer formation. Examples discussed in the present review include the association between aneuploidy, proteotoxic stress, abnormal cellular proliferation, and tumorigenesis; the defective proteins' structure and hence function secondary to gene mutations; and the consequent aberrant networks interactions of abnormal protein repertoire in cancer states. Nevertheless, the field is faced with numerous biological and technical challenges as a result of the concepts of cancer heterogeneity, samples variables, and poor study designs. These challenges can be minimized by proper study designs, implementing strict protocols paying attention to every step in the process, establishing robust validation assays, and exploring innovative tools or even combinations of tools. Besides the traditional proteomics techniques that are constantly being advanced, more recent approaches combining proteomics with other technologies such as imaging are unraveling the complexity of the proteomics changes in cancer and are producing data that are thought to be more representing to the* in vivo* situation and tumor environment. Examples of three of the most studied cancers, lung, breast, and ovarian cancers, have been discussed illustrating various perspectives in approaching the subject of cancer biomarkers, the need to standardize and optimize study design, preanalytical and analytical assays components, and strict validation strategies. Overall, common objectives for proteomics studies in cancer are to better understand tumor biology, to facilitate the development of biomarkers and, most importantly, to move towards bedside applications in cancer management. Refining the huge amount of information obtained from proteomics and related technologies is required to enable transition to clinical validation, which is an ultimate goal for many proteomics-centered studies.
